# Telomere Length and Survival of Patients with Hepatocellular Carcinoma in the United States

**DOI:** 10.1371/journal.pone.0166828

**Published:** 2016-11-23

**Authors:** Baiyu Yang, Fatma M. Shebl, Lawrence R. Sternberg, Andrew C. Warner, David E. Kleiner, Daniel C. Edelman, Allison Gomez, Casey L. Dagnall, Belynda D. Hicks, Sean F. Altekruse, Brenda Y. Hernandez, Charles F. Lynch, Paul S. Meltzer, Katherine A. McGlynn

**Affiliations:** 1 Division of Cancer Epidemiology and Genetics, National Cancer Institute, Bethesda, MD, 20892, United States of America; 2 Yale University School of Public Health, New Haven, CT, 06520, United States of America; 3 Pathology-Histotechnology Laboratory, Frederick National Laboratory for Cancer Research, Frederick, MD, 21701, United States of America; 4 Center for Cancer Research, National Cancer Institute, Bethesda, MD, 20892, United States of America; 5 Cancer Genomics Research Laboratory, Leidos Biomedical Research, Inc. Frederick National Laboratory for Cancer Research, Frederick, MD, 20892, United States of America; 6 Division of Cancer Control & Population Sciences, National Cancer Institute, Bethesda, MD, 20892, United States of America; 7 University of Hawaii Cancer Center, Honolulu, HI, 96813, United States of America; 8 University of Iowa College of Public Health, Iowa City, IA, 52242, United States of America; University of Navarra School of Medicine and Center for Applied Medical Research (CIMA), SPAIN

## Abstract

**Background:**

Telomere shortening is an important molecular event in hepatocellular carcinoma (HCC) initiation; however, its role in HCC progression and prognosis is less clear. Our study aimed to examine the association of telomere length with survival of patients with HCC.

**Methods:**

We measured telomere length in tumor and adjacent non-tumor tissues from 126 persons with HCC in the United States (U.S.) who were followed for mortality outcomes. Relative telomere length (RTL) was measured by a monochrome multiplex quantitative polymerase chain reaction assay. Multivariable Cox proportional hazards modeling was used to calculate hazard ratios (HRs) and 95% CIs for the association between telomere length and all-cause mortality. We also examined associations between telomere length and patient characteristics using multiple linear regression.

**Results:**

During a mean follow-up of 6.0 years, 79 deaths occurred among 114 individuals for whom survival data were available. The ratio of RTL in tumor relative to non-tumor tissue was greater for individuals with regional or distant stage tumors (0.97) than localized stage tumors (0.77), and for individuals with grade III or IV tumors (0.95) than grade II (0.88) or grade I (0.67) tumors. An RTL ratio ≥1 was not associated with survival (HR 0.92, 95% CI 0.55, 1.55) compared to a ratio <1, after adjusting for age at diagnosis, sex, tumor stage and tumor size. Similarly, RTL in the tumor and non-tumor tissue, respectively, were not associated with survival.

**Conclusions:**

This U.S. based study found that telomeres may be longer in more aggressive HCCs. There was no evidence, however, that telomere length was associated with survival of patients with HCC. Future investigations are warranted to clarify the role of telomere length in HCC prognosis.

## Introduction

Hepatocellular carcinoma (HCC) is the second leading cause of cancer mortality worldwide [1]. The prognosis of HCC is very poor, especially for those diagnosed at advanced stages [1]. In the United States (U.S.), where HCC incidence and mortality have been rising rapidly in recent years [2–4], the 5-year survival rate is <20% [5]. It is important to further understand the molecular basis of HCC and identify molecular biomarkers that could serve as potential therapeutic targets.

Telomeres consist of protein-bound tandem repeats of the TTAGGG sequence that form the ends of eukaryotic chromosomes [6]. Telomeres shorten with each cell division due to the “end-replication problem”, and telomere shortening beyond a critical length triggers chromosomal instability and loss of cell viability [6]. Telomere-dependent chromosomal instability may contribute to HCC initiation [7], and several human studies have shown that telomeres are shorter in HCC compared to paired non-tumor tissue [8–18]. However, the role of telomere shortening in HCC prognosis might be different from that in HCC initiation. It has been proposed that HCC progression requires stabilization of telomere length [19] by the activation of telomerase [7] or telomerase-independent mechanisms (i.e., ALT, alternative lengthening of telomeres) [20], which could rescue short telomeres and sustain tumor growth [7]. In telomerase-deficient murine models, it has been reported that telomere shortening suppresses HCC progression [21, 22]. However, very few human studies have investigated the role of telomere length in the prognosis of HCC.

To address these gaps in the literature, we measured relative telomere length (RTL) in tumor and adjacent non-tumor tissue samples from 126 persons with HCC in the U.S., and examined whether telomere length was associated with all-cause mortality after HCC diagnosis. As a secondary aim, a cross-sectional assessment was made on the associations of telomere length with patients’ demographics and tumor characteristics.

## Methods

### Study participants

Our study included 126 individuals diagnosed with HCC in Iowa (n = 99), Hawaii (n = 4), Connecticut (n = 11), and other states in the U.S. (Virginia, Ohio, and Tennessee, n = 12). Persons with HCC from Iowa and Hawaii were identified via the Surveillance Epidemiology and End Results (SEER) Cancer Registry in each state and tissues were obtained from the corresponding SEER Residual Tissue Repository. Persons with HCC from Connecticut were identified via Yale New Haven Hospital Records. Persons from other states were identified through the Corporative Human Tissue Network. The year of diagnosis in our study population ranged from 1973 to 2012. Diagnosis of all cases was confirmed by review of pathological report and review of tissue samples by a liver cancer pathologist at the National Cancer Institute, Bethesda, MD. Only primary HCC cases with available tumor and adjacent normal tissue were included. Individuals who had started therapy prior to donating tissue were not included. We retrieved information on patient demographics, tumor characteristics, limited treatment data, length of survival, and mortality outcomes from the SEER registries for cases from Iowa and Hawaii, and from Yale New Haven Hospital records for cases from Connecticut. For cases from other states, we obtained information on demographics and tumor characteristics from pathology reports, but data on survival were not available for these cases.

The study protocols were approved by the Institutional Review Boards at the National Cancer Institute, the University of Hawaii, the University of Iowa, and Yale University.

### Laboratory procedures

Representative H&E stained slides from the blocks comprising each case were scored for tumor/normal content. Six, ten-micron sections containing varying dimensions of processed tissue were then cut and collected either en masse or first mounted onto Superfrost plus glass slides to facilitate target enrichment by hand macro dissection. 175 uL of microwave retrieval solution (0.1% guanidium thiocyanate and 0.1M NaOH,) was added to the pooled tumor and normal tissue dissectates from each case and heated to 100°C for 10 minutes followed by an additional 10 minutes at 100°C using the microwave module of a Milestone RH-1 microwave tissue processor (Hacker Instruments and Industries, Inc.) [23]. Tubes were then transferred to a Thermomixer R (Eppendorf, North America) for an additional 5 minutes at 65°C and centrifuged for 5 minutes at 14,000g at 4°C. Supernatants were transferred to fresh tubes leaving behind the solidified paraffin. DNA was isolated using the phenol-based AutoGenprep 245T DNA extraction kit according to the provided method. Resultant DNA was re-suspended in 10 mM Tris, pH 8.0. Yield and purity were determined by NanoDrop 1000 spectrophotometer (NanoDrop technologies). DNA stocks were then stored at -80°C until subsequent analysis.

RTL was measured by monochrome multiplex quantitative polymerase chain reaction (MMQPCR) [24] using a non-homologous primer set for the telomere repeats and a GC-clamp primer set for the single copy gene (SCG) albumin. The differences in melting temperature between telomere and albumin amplicons allowed two successive fluorescent readings at two different temperatures using a single DNA binding dye (Syto-82, Invitrogen) in a single tube reaction to differentiate copy number of the targets [25]. Threshold cycles (Ct) and standard curves were established using the Corbett Rotor-Gene 6 automated software with default settings. The telomere and SCG albumin standard curves covered two logs of dynamic range and were generated using normal female DNA (Promega #G152). A sample’s telomere to SCG (T/S) ratio was quantified using the relative ng of telomere and ng of albumin acquired from the telomere and albumin standard curves respectively. This T/S value is a measure of the relative telomere length (RTL) and is expected to be proportional to the average telomere length per cell [24, 26]. Tumor and adjacent non-tumor samples from HCC cases were each tested in triplicate, and T/S values were averaged. For each individual, we also calculated the ratio of RTL between tumor and paired non-tumor samples (hereafter referred to as RTL ratio).

All samples were measured by laboratory personnel who were blinded to the status of the tissue (tumor vs. non-tumor). The coefficient of variation (CV) of the C_t_ for telomere and SCG triplicate assays were 0.47% and 0.34%, respectively. In addition, the variability of the RTL ratio was assessed using quality control samples. The CV of the RTL ratio of quality control samples was 12.3% within plate (based on four standard samples within each plate), and 8.1% between plates (based on one positive control sample repeatedly measured across all plates).

### Statistical analysis

We used the Wilcoxon signed rank test to compare the RTL between tumor and paired non-tumor tissue samples. We tested whether RTL in the tumor and non-tumor tissue, as well as the RTL ratio, differ by age at diagnosis, sex, geographic area, race, and tumor characteristics at diagnosis (SEER summary stage, size, and grade) using multiple linear regression. Because the RTL and RTL ratio were not normally distributed, all values were log-transformed before this analysis, and geometric means were calculated.

To assess the association between telomere length and all-cause mortality among individuals with HCC, we categorized the RTL ratio using a cut-off point of 1: an RTL ratio <1 means the tumor tissue has shorter telomere relative to the paired non-tumor tissue, and an RTL ratio ≥1 indicates the tumor tissue has equal or longer telomere relative to the paired non-tumor tissue. We also examined the RTL ratio, as well as RTL in tumor and non-tumor tissues, respectively, in continuous form. We used Cox proportional hazard modeling to calculate the hazard ratios (HRs) and 95% CIs, using time since diagnosis as the underlying time metric. Person-time began on the date of diagnosis and ended on either death or last date of follow-up (June 2015 for cases from Iowa, November 2013 for cases from Hawaii, and last hospital visit for cases from Connecticut), whichever came first. We evaluated the proportional hazards assumption for the main exposures using a likelihood ratio test by comparing models with and without an interaction term between the exposure and log time; no violations were detected. For the multivariate models, we adjusted for age at diagnosis, sex, tumor stage at diagnosis, and tumor size at diagnosis as these variables changed the HR estimate by more than 10%. Other variables were evaluated but not included in the final model, including geographic area, race, and tumor grade.

All analyses were conducted using SAS version 9.3 (SAS Institute, Cary, NC). All statistical tests were two-sided, and p values < 0.05 were considered statistically significant. For analyses involving RTL in the tumor or paired non-tumor tissue, we calculated batch-adjusted RTL as the RTL of the study sample divided by that of the positive control sample on each plate. For analyses involving the RTL ratio, no batch-adjustment was made as paired tumor and non-tumor tissue samples were placed within the same plate.

## Results

Basic characteristics of the 126 HCC cases in our study are shown in Table 1. The mean age at diagnosis was 61.2 years (standard deviation 14.3 years), 64% were men, and 74% had localized stage tumors at diagnosis. The majority of the cases (90%) were white, and 79% were retrieved through the cancer registry in Iowa. Overall, the HCC tissues had statistically significantly shorter RTL than their matched non-tumor tissues (p < 0.01, Wilcoxon signed rank test). The RTL ratio comparing tumor to non-tumor tissue was <1 in 89 pairs (71%), and ≥1 in 37 pairs (29%).

**Table 1:** Characteristics of hepatocellular carcinoma cases (N = 126).

Characteristic	
Age at diagnosis (years), mean (SD)	61.2 (14.3)
Sex, N (%)	
Men	81 (64.3)
Women	45 (35.7)
Geographic area, N (%)	
Iowa	99 (78.6)
Hawaii	4 (3.2)
Connecticut	11 (8.7)
Other U.S. states (VA, TN, OH)	12 (9.5)
Race, N (%)	
White	113 (89.7)
Black	4 (3.2)
Other	8 (6.4)
Stage at diagnosis, N (%)	
Localized	93 (73.8)
Regional	28 (22.2)
Distant	5 (4.0)
Grade at diagnosis, N (%)	
Grade I	30 (23.8)
Grade II	51 (40.5)
Grade III	21 (16.7)
Grade IV	2 (1.6)
Tumor size at diagnosis, N (%)	
< 5cm	61 (48.4)
≥ 5cm	63 (50.0)
RTL in tumor tissue, median	0.48
RTL in non-tumor tissue, median	0.58
RTL tumor/non-tumor ratio, median	0.75
RTL tumor/non-tumor ratio categories, N (%)	
<1	89 (70.6)
≥1	37 (29.4)

As shown in Table 2, the RTL tumor/non-tumor ratio was greater for tumors with more advanced stage or higher grade (p = 0.03 for both). RTL in the tumor was also longer for tumors with more advanced stage (p = 0.01). RTL in the non-tumor tissue was shorter among older individuals (p <0.01). In addition, we observed differences in tumor and non-tumor tissue RTL across geographic areas; however, the sample size was small for some areas.

**Table 2:** Association of relative telomere length with demographic factors and tumor characteristics.

		RTL in tumor tissue^1^			RTL in non-tumor tissue^1^			RTL ratio (tumor/non-tumor)^1^		
	N	Mean	95% CI	P value	Mean	95% CI	P value	Mean	95% CI	P value
**Age at diagnosis**				0.42			<0.01			0.35
<53	30	0.62	0.44, 0.89		0.72	0.61, 0.85		0.79	0.60, 1.05	
53-<61	31	0.50	0.35, 0.71		0.65	0.55, 0.77		0.73	0.56, 0.96	
61-<73	33	0.53	0.39, 0.73		0.55	0.47, 0.63		0.92	0.72, 1.18	
≥73	32	0.49	0.34, 0.70		0.52	0.45, 0.62		0.85	0.64, 1.13	
**Sex**				0.87			0.15			0.79
Men	81	0.53	0.39, 0.71		0.58	0.51, 0.66		0.85	0.68, 1.06	
Women	45	0.54	0.39, 0.74		0.65	0.56, 0.75		0.83	0.64, 1.07	
**Geographic area**				<0.01			<0.01			0.49
Iowa	99	0.47	0.38, 0.57		0.55	0.51, 0.59		0.84	0.67, 1.04	
Hawaii	4	0.29	0.15, 0.55		0.29	0.20, 0.41		1.10	0.63, 1.92	
Connecticut	11	0.72	0.47, 1.10		0.72	0.58, 0.89		1.00	0.68, 1.47	
Other U.S. states (VA, TN, OH)	12	0.81	0.54, 1.22		1.19	0.97, 1.46		0.77	0.53, 1.12	
**Race**				0.12			0.05			0.34
White	113	0.47	0.35, 0.64		0.58	0.52, 0.66		0.82	0.65, 1.02	
Black	4	0.77	0.41, 1.47		0.86	0.60, 1.24		1.05	0.62, 1.78	
Other	8	0.67	0.41, 1.08		0.69	0.54, 0.89		1.02	0.67, 1.56	
**Tumor stage**				0.01	N/A					0.03
Localized	93	0.45	0.36, 0.55					0.77	0.64, 0.91	
Regional or distant	33	0.62	0.46, 0.83					0.97	0.78, 1.20	
**Tumor size**				0.56	N/A					0.67
<5cm	61	0.56	0.40, 0.77					0.86	0.67, 1.11	
≥5cm	63	0.52	0.39, 0.70					0.83	0.66, 1.04	
**Tumor grade**				0.60	N/A					0.03
Grade I	30	0.44	0.31, 0.63					0.67	0.52, 0.86	
Grade II	51	0.53	0.37, 0.75					0.88	0.72, 1.08	
Grade III or IV	23	0.48	0.33, 0.72					0.95	0.74, 1.23	

Abbreviations: RTL, relative telomere length.

^1^Geometric means presented for all variables. Models for RTL in tumor adjusted for tumor stage and geographic area, models for RTL in non-tumor tissue adjusted for age and geographic area, and models for the RTL ratio adjusted for tumor stage and grade, as appropriate.

During a mean follow-up of 6.0 years, 79 deaths occurred among 114 individuals from whom survival information was available. We observed no associations of RTL ratio with survival (Table 3). Compared to individuals with RTL ratio <1, the HR for those with RTL ratio ≥1 was 0.92 (95% CI 0.55, 1.55) for all-cause mortality, after adjusting for age at diagnosis, sex, tumor stage and tumor size. Similarly, no association was observed when we categorized the study population using the median split of RTL ratio, or when we examined it in continuous form. Adjusted HR results were also null when we examined RTL in tumor and paired non-tumor tissues, respectively, using median split or the continuous form (data not shown).

**Table 3:** Association of telomere length with survival among hepatocellular cancer cases.

	No. of deaths/No. at risk	Person-years	Crude HR (95% CI)	Adjusted HR (95% CI)^1^
RTL tumor/non-tumor ratio				
<1	51/80	541.0	Ref	Ref
≥1	28/34	146.8	1.80 (1.13, 2.87)	0.92 (0.55, 1.55)
< median^2^	36/57	410.3	Ref	Ref
≥ median^2^	43/57	277.5	1.53 (0.97, 2.40)	1.05 (0.65, 1.69)
Continuous, per unit increase	N/A	N/A	1.81 (1.22, 2.69)	1.18 (0.74, 1.88)

Abbreviations: HR, hazard ratio; RTL, relative telomere length.

^1^Models adjusted for age at diagnosis, sex, tumor stage at diagnosis, and tumor size at diagnosis.

^2^Median value is 0.78 among individuals with data on survival time.

## Discussion

To our knowledge, this study is the first to examine RTL in HCC tissue samples among HCC patients in the U.S., historically a low-risk area where HCC incidence has been rising rapidly in recent years. We found that the RTL tumor/non-tumor ratio were longer for tumors with more advanced stage and higher grade. However, there was no evidence for an association between telomere length and survival among persons with HCC.

Telomere shortening may be an important molecular event in HCC initiation. A number of human studies have reported that telomeres are shorter in HCC compared to paired non-tumor tissue [8–18], consistent with our findings. Studies have also found that telomere shortening occurs in chronic liver disease [9, 27] and dysplastic nodules [14], suggesting that telomere shortening is a critical event in the multistep process that leads to HCC. However, the role of telomere shortening in HCC progression and prognosis might be different from that in HCC initiation. Tumor progression requires stabilization of telomeres [19] by the activation of telomerase [7] or telomerase-independent mechanisms (i.e., ALT) [20], which may rescue short telomeres and sustain tumor growth [7]. In murine models, telomere shortening in the absence of telomerase activity suppresses the progression of HCC [21, 22]. In addition, in vitro HCC studies have shown that longer telomeres are associated with greater invasive capacity [28]. Most human cancer metastases contain telomerase-positive cells [6], indicating the role of telomere maintenance in tumor progression. However, population-level evidence on the associations of telomere length and telomere maintenance mechanisms with HCC survival is scarce.

In this study, we examined the association between telomere length and survival among HCC patients. Although longer telomere length was associated with increased mortality in crude analyses, no associations persisted after we adjusted for important clinical variables such as tumor stage. Previously, three studies have examined telomere length in relation to survival of HCC [16, 29, 30]. Among 49 HCC cases from Korea, individuals with higher tissue RTL tumor/non-tumor ratio experienced poorer survival [16], however this analysis was not adjusted for covariates. Two studies of HCC patients from China revealed that longer peripheral blood leukocyte RTL was associated with poorer overall survival [29, 30]. In addition to telomere length, a few studies found that higher telomerase activity was associated with poorer survival [16] or higher risk of recurrence [31]. Also, a Korean study reported that the telomerase reverse transcriptase (TERT) promoter methylation was associated with higher TERT expression and poorer survival among HCC patients [32]. Collectively, the current evidence suggests an association of longer telomere or activation of telomere maintenance with poor prognosis of HCC, however most of the previous studies have a relatively small sample size and some did not adjust for important confounders. Moreover, all the studies were conducted in high-risk areas of HCC such as Asia. Since etiology of HCC in the U.S. (a low-risk area) may be different from that in high-risk areas [33], the prognostic value for a particular biomarker might differ as well [34]. Therefore, future studies among low-risk regions are particularly needed to examine the role of telomere length in HCC survival.

Our study suggests that the RTL tumor/non-tumor ratio is longer in tumors with advanced stage or higher grade. Several previous studies also observed longer telomeres and greater telomerase activity in HCC tumors with poor differentiation [16, 31], larger size [35], or advanced stage [16]. Although it is not possible to establish the temporality between telomere maintenance and tumor progression in cross-sectional studies, these observations are consistent with the hypothesis that the maintenance of telomere may be necessary to sustain tumor growth [7]. Specifically, we speculate that ALT [36] may explain the elongated telomeres observed in more aggressive tumors in our study, especially for subjects whose RTL ratio >1, as it is not biologically plausible for telomerase to result in such substantial elongation of telomeres [37], whereas ALT cells demonstrate highly heterogeneous telomere length, some of which are extremely long [38]. Future studies testing for ALT are warranted to further clarify the role of telomerase-independent telomere maintenance mechanisms in HCC progression.

A major strength of this study is that it is the first study to evaluate tissue telomere length in HCCs from the U.S., a non-high-risk region. We confirmed previous findings that telomeres are shorter in tumor compared to adjacent non-tumor tissues, and further explored the associations of telomere length with patient characteristics and all-cause mortality. Given that the etiology [33] and prognosis [34] of HCC differ in high-risk and low-risk areas, our study is a valuable addition to previous reports that were conducted in high-risk areas. In addition, our study measured RTL by the novel MMQPCR method, which minimizes any variation from pipetting [24]. However, there are several limitations in our study. We lacked data on telomerase activity and ALT, which might provide insight into the role of telomere maintenance in HCC prognosis. Given that the patients’ year of diagnosis ranged from 1973 to 2012, it is likely that treatment plans have changed over the years; however, we lacked detailed treatment data, and we were unable to stratify the survival analysis by year of diagnosis due to the relatively small sample size. In addition, we lacked information on HCC etiology so could not examine differences in RTL by major risk factors or evaluate these risk factors as potential confounders in the survival analysis.

In conclusion, our study suggests that telomere may be longer in tumors with more advanced stage or higher grade, consistent with the hypothesis that telomere length restoration may be involved in tumor progression. However, we observed no association between telomere length and overall survival of patients with HCC. Future studies are needed to comprehensively evaluate telomere length and telomere maintenance mechanisms in relation to HCC prognosis, especially among a low-risk population.

## Supporting Information

S1 File Analytical dataset. (SAS7BDAT)

## Abbreviations

ALT: alternative lengthening of telomeres
C_t_: threshold cycle
CV: coefficient of variation
HCC: hepatocellular carcinoma
HR: hazard ratio
MMQPCR: monochrome multiplex quantitative polymerase chain reaction
RTL: relative telomere length
SCG: single copy gene
SEER: Surveillance Epidemiology and End Results
TERT: telomerase reverse transcriptase
T/S: telomere to single copy gene
U.S.: United States
